# A potential gliovascular mechanism for microglial activation: differential phenotypic switching of microglia by endothelium versus astrocytes

**DOI:** 10.1186/s12974-018-1189-2

**Published:** 2018-05-15

**Authors:** Changhong Xing, Wenlu Li, Wenjun Deng, MingMing Ning, Eng H. Lo

**Affiliations:** 1 Neuroprotection Research Laboratory, Departments of Radiology and Neurology, Massachusetts General Hospital, Harvard Medical School, MGH East 149-2401, Charlestown, MA 02129 USA; 2 Department of Neurology, Massachusetts General Hospital, Harvard Medical School, Boston, MA 02114 USA

**Keywords:** Microglia, Endothelium, Astrocyte, Phenotypic switching, Biphasic

## Abstract

**Background:**

Activation of microglia can result in phenotypic and functional diversity. However, the pathways that trigger different states of microglial activation remain to be fully understood. Here, we hypothesized that after injury, astrocytes and endothelium may contribute to a gliovascular switch for microglial activation.

**Methods:**

Astrocytes or cerebral endothelial cells were subjected to oxygen glucose deprivation, then conditioned media were transferred to microglia. The release of TNFα, IL-1β, IL-10, and IGF-1 was measured using ELISA. Surface markers of CD11b, CD45, CD86, and MHC class II were detected by flow cytometry. mRNA expression of iNOS, CD86, CD206, Arginase1, and transcription factors was measured using real-time PCR. Microglial function including migration and phagocytosis was assessed. Dendritogenesis was determined by counting the number of primary dendrites, secondary dendrites, and dendritic ends in the neurons exposed to either endothelial- or astrocyte-activated microglia.

**Results:**

Exposure to conditioned media from oxygen-glucose-deprived cerebral endothelial cells or oxygen-glucose-deprived astrocytes activated microglia into different forms. The endothelium converted ramified microglia into amoeboid shapes; increased the release of TNFα, IL-1β, and IL-10; decreased IGF-1; upregulated iNOS expression; and inhibited microglial migration and phagocytosis. In contrast, astrocytes increased microglial production of IGF-1, upregulated CD206 expression, and enhanced microglial phagocytosis. These opposing effects of the endothelium versus astrocyte crosstalk partly mirror potentially deleterious versus potentially beneficial microglial phenotypes. Consistent with this idea, endothelial-activated microglia were neurotoxic, whereas astrocyte-activated microglia did not affect neuronal viability but instead promoted neuronal dendritogenesis.

**Conclusion:**

These findings provide proof of concept that endothelial cells and astrocytes provide differing signals to microglia that influence their activation states and suggest that a gliovascular switch may be involved in the balance between beneficial versus deleterious microglial properties.

**Electronic supplementary material:**

The online version of this article (10.1186/s12974-018-1189-2) contains supplementary material, which is available to authorized users.

## Background

Microglia are sentinels of the brain. They constantly monitor the microenvironment and actively respond to injury and disease [1–4]. Any insult to the central nervous system (CNS), including infection, trauma, or metabolic dysfunction, causes microglial activation. Activation of microglia can result in phenotypic and functional diversity—harmful or beneficial—depending on the activating conditions.

Traditionally, “M1-like” microglia are thought to be damaging by releasing free radicals and cytokines that amplify inflammation and secondary neuronal death [5–7]. However, beneficial “M2-like” microglia may also exist that promote CNS recovery by secreting neurotrophic factors [8, 9], promoting synaptic plasticity [10], and supporting remyelination [11] and angiogenesis [12]. Activated microglia play a dual role in ischemic stroke. Activated microglia can be neurotoxic by releasing reactive oxygen species and pro-inflammatory cytokines [13–17]. On the other hand, the accumulation of microglia may correlate with the reduction of neuronal damage under some conditions [18–21]. The neurotoxic and neuroprotective subpopulations of microglia coordinate the inflammatory process, and the predominance of one or the other would result in either more damage or attempted recovery [22]. These potentially biphasic and overlapping properties of microglia are well accepted, but the complex mechanisms that trigger and regulate these different states of activation remain to be fully dissected [23, 24].

The concept of the neurovascular unit suggests that interactions between all cell types in the CNS mediate the pathophysiology of stroke, brain injury, and neurodegeneration [25]. More recently, the concept of help-me signaling suggests that the balance between injury and repair in the CNS may be mediated in part by crosstalk between multiple cells of the neurovascular unit [26–28]. Is it possible that different states of microglial activation may also depend on signaling from other CNS cell types? In the context of stroke, two candidate “partners” for microglial crosstalk and activation might comprise the endothelium and astrocyte, because these cells respond quickly and profoundly to cerebral ischemia [29, 30]. Here, we propose a gliovascular mechanism that regulates the microglial switch, i.e., differential signaling from reactive endothelium or reactive astrocytes triggers different states of microglial activation that may in turn influence neuronal viability.

## Methods

### Microglia

Primary microglia cultures were prepared from the cerebral cortices of 1-day-old neonatal Sprague-Dawley rats according to the previously described standard methods [31, 32]. After removing the meninges, cortical tissues were digested with 0.25% trypsin-EDTA for 30 min at 37 °C, followed by mechanical triturating in DMEM/F12 with 10% fetal bovine serum (FBS). The mixed cortical cells were plated in DMEM/F12 with 10% FBS, and the medium was completely replaced every 3–4 days and confluency was achieved after about 14 days in vitro (DIV). After then, the microglia were isolated from mixed glial cultures via mild trypsinization. Incubation of mixed glial cultures with a trypsin solution (0.25% trypsin-EDTA diluted 1:4 in DMEM/F12) for 15–25 min resulted in the detachment of an intact layer of cells in one piece, whereas microglial cells remained attached to the bottom of the well. Microglia were allowed to rest overnight prior to treatments. Microglia were treated with conditioned media from astrocytes or endothelial cells for various times.

### Astrocytes

Primary astrocyte cultures were prepared from the cerebral cortices of 1-day-old neonatal Sprague-Dawley rats using standard methods, as previously described [33]. Briefly, the dissociated cortical cells were suspended in DMEM containing 10% FBS and plated in 75-cm^2^ flasks. After 10–14 DIV, astrocytes were obtained from the mixed glial cultures by shaking at 220 rpm overnight to remove microglia and oligodendrocytes. Astrocytes were dissociated by trypsinization and then reseeded on collagen-coated plates. Passages 2 to 4 of primary cultured astrocytes were used for experiments.

### Cerebral endothelial cells

RBE.4 cells, a rat brain endothelial cell line, were cultured in flasks coated with rat tail collagen I (Corning, Bedford, MA) and maintained in endothelial basal medium-2 (Lonza, Hopkinton, MA) supplemented with FBS, fibroblast growth factor-2, epidermal endothelial growth factor, hydrocortisone, insulin-like growth factor, ascorbic acid, VEGF, and amphotericin B.

### Neurons

Primary hippocampal neuron cultures were prepared using standard methods, as previously described [34]. Briefly, the hippocampus was dissected out from the embryonic Sprague-Dawley rat fetuses E19, then dissociated, both enzymatically (0.25% trypsin-EDTA) for 15 min at 37 °C and mechanically. The single-cell suspension was diluted in serum-free neurobasal medium containing 2% B27 supplement and 0.5 mM l-glutamine and then seeded onto pre-coated plates with poly-d-lysine at 3 × 10^5^ cells/ml. The medium was half renewal every 3 days. Neuronal cultures were used for experiments on 8–10 days after seeding.

### Oxygen-glucose deprivation

Oxygen-glucose deprivation (OGD) experiments were performed using a humidified incubator chamber kept at 37 °C, which contained an anaerobic gas mixture (90% nitrogen, 5% hydrogen, and 5% carbon dioxide). To initiate OGD, the culture medium was replaced with no glucose DMEM. After OGD, the cultures were removed from the anaerobic chamber, and the medium was replaced with DMEM/F12 with 10% FBS in a regular incubator. Astrocytes or RBE.4 cells were subjected to OGD for 4 h, and conditioned media from astrocytes or RBE.4 cells were collected 24 h after reoxygenation.

### ELISA

Microglia were treated with endo-CM or astro-CM for 24 h, and then the releases of TNFα, IL-1β, IL-10, and IGF-1 from endo-CM-activated microglia versus astro-CM-activated microglia were measured. TNFα (eBioscience, San Diego, CA), IL-1β (R&D), IL-10 (eBioscience), and IGF-1 (R&D) in cell culture supernatant were measured using ELISA kits, according to manufacturer’s instructions.

### Flow cytometry

Microglial cells were treated with the conditional media from OGD-treated endothelial cells or astrocytes for 24 h. Cells were stained with fluorochrome-conjugated antibodies recognizing cell surface antigens, including CD11b, CD45, CD86, and MHC class II at 4 °C for 30 min. The expression levels of these markers were detected by flow cytometer.

### Real-time PCR

Total RNAs were extracted from cultured microglial cells treated with conditioned media from OGD-treated endothelial cells or astrocytes for various times. For the analysis of mRNA expression of iNOS, CD86, CD206, Arginase1, IRF4, IRF5, IRF7, IRF8, IRF9, STAT1, STAT3, and STAT6, real-time PCR was carried out using TaqMan gene expression assays (Applied Biosystems, Carlsbad, CA), and relative expression was calculated using the 2^−ΔΔCt^ method with normalization to hypoxanthine phosphoribosyltransferase 1 and β-2-microglobulin. All real-time PCRs were performed in triplicates.

### Microglia function

Cell migration assay kit (Chemicon, Billerica, MA) was used to analyze microglial migration. Microglia suspension was loaded into the culture well inserts (membrane pore size of 5 μm) and placed in 24-well plates containing serum-free DMEM/F12 media in the presence or absence of the conditional media from OGD-treated endothelial cells or astrocytes. After incubating for 4 h, the migrated cells were detached from the bottom of the insert and lysed and stained using the CyQUANT^®^ GR Dye. The samples were read using a fluorescence microplate reader set up at 480 nm excitation and 520 nm emission. Migrated cell number was determined by running a fluorescent cell dose curve. To assess phagocytosis, microglial cells were seeded to a 96-well plate at a concentration of 1.0 × 10^5^ cells/well and incubated with or without the conditional media from OGD-treated endothelial cells or astrocytes for 24 h. Then, the cells were incubated with fluorescein-labeled *Escherichia coli* K-12 BioParticles (Invitrogen, Grand Island, NY) for 2 h at 37 °C. Cells were rinsed with 0.25 mg/ml trypan blue to quench extracellular fluorescence. Intracellular fluorescence was read using a fluorescence microplate reader set up with excitation at 480 nm and emission at 520 nm. The experiments were performed with five replicates per condition and repeated a minimum of three times.

### Dendritogenesis

At 8 DIV, the hippocampal neurons were transfected with pEGFP-C1 vector. Then, conditioned media from OGD-treated astrocytes (astro-CM) or astro-CM-treated microglia (astro-CM-treated MCM) were added to the neuron cultures for 5 days. The photos of transfected neurons were taken using Nikon microscope, and the number of primary dendrites, secondary dendrites, and dendritic ends was counted. The complexity of dendritic arbors was calculated with dendritic ends divided by primary dendrites.

### Statistical analysis

Data were expressed as mean ± SD or SEM. Three to five separate experiments were performed for measurements. Data were analyzed using one-way ANOVA (SPSS 21) or *t* test. Statistical significance was set at *p* < 0.05.

## Results

### Different effects of oxygen-glucose-deprived endothelium versus astrocytes on microglial activation

Rat cerebral endothelial cells and astrocytes were subjected to oxygen-glucose deprivation (OGD) for 4 h followed by 24 h of reoxygenation. As expected, endothelial cells and astrocytes were relatively resistant to OGD, and cell viability was not significantly affected (Additional file 1: Figure S1). Then, we transferred OGD-injured endothelial-conditioned media (endo-CM) and OGD-injured astrocyte-conditioned media (astro-CM) onto the microglia to assess the overall hypothesis that was after brain injury, endothelium and astrocytes can shift microglial activation in different ways.

**Fig. 1:**
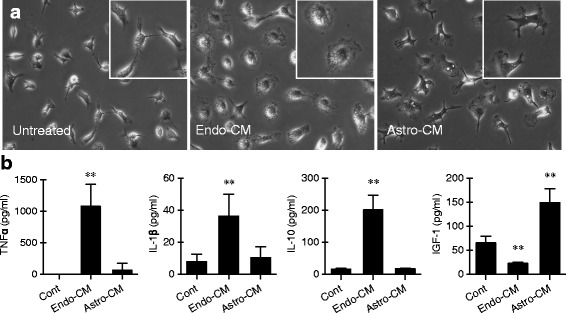
Different effects of oxygen-glucose-deprived endothelium versus astrocytes on microglial activation. **a** Morphology of primary microglia without or with 24 h of treatment with conditioned media from the OGD-treated cerebral endothelial cells or astrocytes. Untreated “resting” microglia displayed a ramified morphology (left). Endo-CM treatment activated microglia into an amoeboid appearance (middle). Astro-CM treatment led to microglial cell body enlargement and reduced peripheral branching (right). **b** Secretion of TNFα, IL-1β, IL-10, and IGF-1 from the primary microglia treated with conditioned media from OGD-treated cerebral endothelial cells or astrocytes for 24 h were measured using ELISA. Endothelial-activated microglia significantly increased the production of TNFα, IL-1β, and IL-10 and reduced IGF-I release. In contrast, astrocyte-activated microglia did not alter the levels of TNFα, IL-1β, and IL-10 but significantly increased the IGF-1 release. Data were expressed as mean ± SD. **p < 0.01 compared to the control (one-way ANOVA). *n* = 3 to 5 independent experiments

First, we observed the microglial morphology under the different treatment conditions. Untreated “resting” microglia displayed a ramified morphology (Fig. 1a, left). Endo-CM treatment activated microglia into an amoeboid appearance (Fig. 1a, middle). Astro-CM treatment led to microglial cell body enlargement and reduced peripheral branching (Fig. 1a, right).

Second, we asked whether these differentially activated microglia show differences in representative cytokines and growth factors. ELISAs were used to quantify the release of TNFα, IL-1β, IL-10, and IGF-1 from endo-CM-activated microglia versus astro-CM-activated microglia. The production of TNFα, IL-1β, and IL-10 were significantly increased in endothelial-activated microglia (Fig. 1b). In contrast, TNFα, IL-1β, and IL-10 were not altered in astrocyte-activated microglia (Fig. 1b). Opposite responses were noted for IGF-1. Endothelial-activated microglia showed a reduction in the IGF-1 release, whereas astrocyte-activated microglia showed an increase in the IGF-1 release (Fig. 1b). Because we used media transfer, it is important to exclude the possibility that these changes indirectly came from endothelial or astrocyte media, instead of directly reflecting microglial responses. Levels of TNFα, IL-1β, IL-10, and IGF-1 in culture media from OGD-treated endothelial cells or astrocytes were measured using ELISA. All the selected cytokines and growth factors were either undetectable or considerably lower (Additional file 2: Figure S2).

**Fig. 2:**
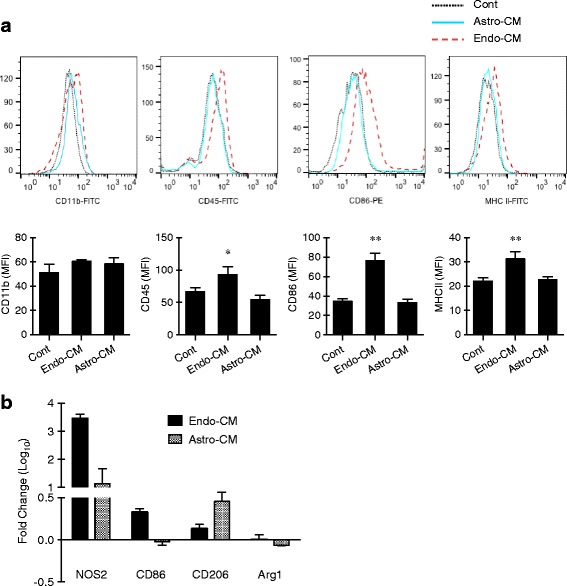
Oxygen-glucose-deprived endothelium and astrocyte activated microglia into different phenotypes. **a** Cell surface markers of microglia were detected by flow cytometry. Expression levels of CD45, CD86, and MHC II were significantly elevated in endo-CM-treated microglia. In contrast, no changes in these cell surface markers were detected in astro-CM-treated microglia. Data were expressed as mean ± SD. *p < 0.05 and **p < 0.01 compared to the control (one-way ANOVA). *n* = 3 independent experiments. **b** Microglial activation markers (iNOS, CD86, CD206, and arginase 1) were measured with real-time PCR. Endo-CM significantly increased microglial expression of iNOS. CD86 was upregulated in endothelial-activated microglia, whereas CD206 was upregulated in astrocyte-activated microglia. Data were expressed as mean ± SEM. *n* = 3 independent experiments

Third, we used flow cytometry to assess the representative cell surface antigens that may reflect different states of microglial activation. Expression levels of CD45, CD86, and MHC II were significantly elevated in endo-CM-treated microglia (Fig. 2a). In contrast, no changes in these cell surface markers were detected in astro-CM-treated microglia (Fig. 2a). Neither endothelial- nor astrocyte-activated microglia showed any response in CD11b (Fig. 2a).

Fourth, we used real-time PCR to further characterize different markers of microglia activation. Both endo-CM and astro-CM treatments significantly increased the microglial expression of iNOS, although this response was much larger in endothelial-activated versus astrocyte-activated microglia (Fig. 2b). CD86 was upregulated in endothelial-activated microglia, whereas CD206 was upregulated in astrocyte-activated microglia (Fig. 2b). No effects of endo-CM or astro-CM were observed for microglial expression of Arg1 (Fig. 2b).

**Fig. 3:**
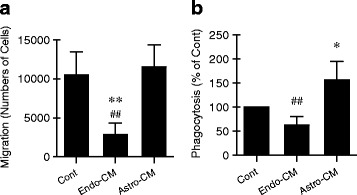
Altered microglia functions after treatment with conditioned media from endothelial cells or astrocytes. **a** Migration. **b** Phagocytosis. Endo-CM treatment inhibited microglial migration and phagocytosis, whereas astro-CM treatment enhanced microglial phagocytosis. Data were expressed as mean ± SD. *p < 0.05 and **p < 0.01 compared to the control; ^##^*p* < 0.01 compared to astro-CM-treated microglia (one-way ANOVA). *n* = 5 to 7 independent experiments

Finally, we asked whether endothelium and astrocytes may also affect microglial function. Endo-CM treatment inhibited microglial migration (Fig. 3a) and phagocytosis (Fig. 3b), whereas astro-CM treatment enhanced microglial phagocytosis (Fig. 3b).

### Different effects of endothelial-activated versus astrocyte-activated microglia on neurons

**Fig. 4:**
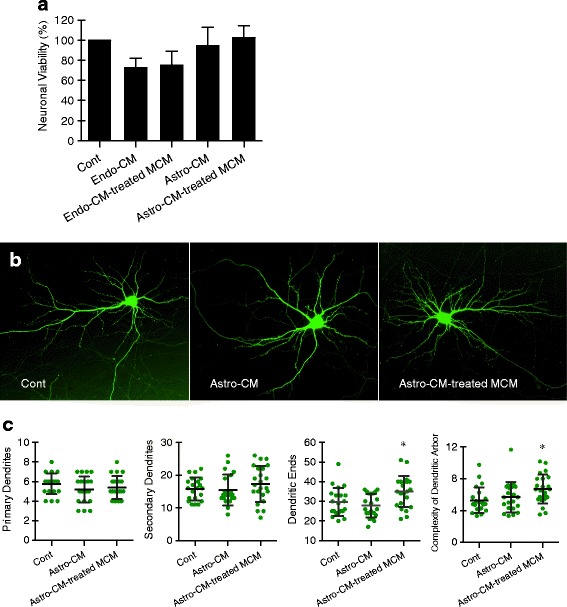
Different effects of endothelial-activated versus astrocyte-activated microglia on the neurons. **a** MTT assays of neuronal cell viability. Conditioned media from endothelial-activated microglia were moderately neurotoxic. In contrast, conditioned media from astrocyte-activated microglia did not affect neuronal viability. *p* = 0.0272 (one-way ANOVA). **b** Representative hippocampal neurons transfected with pEGFP-C1 vector. **c** Graph of dendritogenesis. Astrocyte-activated microglia significantly increased dendritic complexity. Data were expressed as mean ± SD. *p < 0.05 compared to the control (one-way ANOVA). *n* = 21 to 23 individual neurons

Our findings indicated that endothelial cells and astrocytes might activate microglia into different phenotypes. Here, we performed two-step media transfer experiments to test whether these differentially activated microglia were toxic or beneficial towards neurons. First, rat cerebral endothelial cells or astrocytes were subjected to similar protocols of 4 h OGD and 24 h reoxygenation; then, their conditioned media were collected and added to microglia. After 24 h of culture in either endo-CM or astro-CM, a second media transfer was performed, i.e., microglial-conditioned media were collected and transferred onto the primary rat cortical neurons. Neuronal cell viability assays showed that conditioned media from endothelial-activated microglia were moderately neurotoxic (*p* = 0.0272, one-way ANOVA) (Fig. 4a). In contrast, conditioned media from astrocyte-activated microglia did not affect neuronal viability (Fig. 4a).

To assess potential benefit, we next measured the effects of these activated microglia on surrogate markers of neuroplasticity. Once again, a two-step media transfer experiment was performed. The rat cerebral endothelial cells or astrocytes were subjected to 4 h OGD and 24 h reoxygenation, and then their conditioned media were collected and added to microglia. After 24 h of culture in either endo-CM or astro-CM, a second media transfer was performed, i.e., microglial-conditioned media were collected and transferred onto the primary rat hippocampal neurons previously labeled with green fluorescent protein (Fig. 4b). After 5 days, dendritic morphology was quantified. Endothelial-activated microglia did not appear to alter neuronal dendrites. However, astrocyte-activated microglia significantly increased dendritic complexity (Fig. 4b, c).

### Potential roles of transcription factors in microglial phenotypic switching

**Fig. 5:**
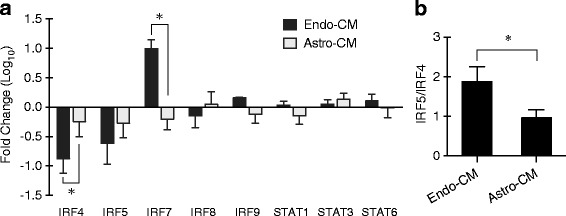
Potential roles of transcription factors in microglial phenotypic switching. **a** Expression of IRFs and STATs were detected by real-time PCR. Endo-CM treatment significantly downregulated IRF4 expression and upregulated IRF7 expression in microglia, but astro-CM treatment did not significantly change the expression levels of IRFs and STATs. Data were expressed as mean ± SD. *p < 0.05 compared to the control (*t* test). *n* = 3 independent experiments. **b** Bar graph of the ratio of IRF5/IRF4. Endo-CM treatment significantly raised the ratio of IRF5/IRF4 in microglia compared with astro-CM treatment. Data were expressed as mean ± SD. *p < 0.05 (*t* test). *n* = 3 independent experiments

To explore the potential roles of transcription factors in microglia phenotypic switch, we detected several members of interferon-regulatory factor (IRF) protein family and the signal transducer and activator of transcription (STAT) protein family using real-time PCR, including IRF4, 5, 7, 8, and 9 and STAT1, 3, and 6. The microglia were incubated with the conditioned media from OGD-treated endothelial cells or astrocytes for 4 h. Endo-CM treatment significantly downregulated IRF4 expression and upregulated IRF7 expression in microglia, but astro-CM treatment did not significantly change the expression levels of IRFs and STATs (Fig. 5a). In addition, endo-CM treatment significantly raised the ratio of IRF5/IRF4 in microglia compared with astro-CM treatment (Fig. 5b).

## Discussion

**Fig. 6:**
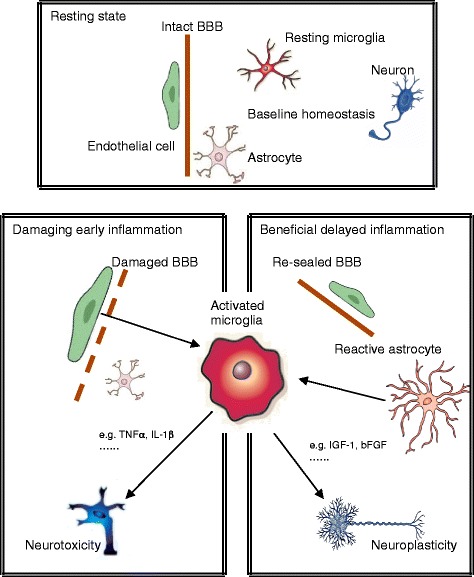
Summary schematic of gliovascular regulation of the microglial switching from injury into repair within the neurovascular unit. The first phase of deleterious microglia matches the loss of BBB integrity during acute injury. The second phase of beneficial microglia matches the appearance of reactive astrocytes during delayed recovery

Injury or disease will trigger complex cascades of multi-cellular and multi-factorial pathways in the CNS. It is now recognized that these endogenous mechanisms comprise a mix of potentially deleterious and potentially beneficial responses. After stroke or brain injury, initial pathways of secondary damage in the acute phase may eventually yield to endogenous attempts at tissue remodeling during the recovery phase [35]. In neurodegeneration, the onset of a disease is initially counterbalanced by repair mechanisms of neuroplasticity and adaptation, but eventually, accumulating deficits overcome the ability to compensate [36]. Many cells will surely be involved, but the dynamic balance between good and bad can perhaps be best reflected in the complex spectrum of microglia activation. All these differing aspects of microglial function are typically swept under a general rubric of activation. But how are different states of microglial activation initially triggered? Our current study suggests that crosstalk with other cells in the neurovascular unit may play a potential role. Conditioned media from OGD-injured cerebral endothelial cells activated microglia and caused them to release large amounts of TNFα and IL-1β; upregulated CD45, CD86, MHCII, and iNOS; and inhibited microglial migration and phagocytosis. In contrast, microglia that were activated by conditioned media from OGD-injured astrocytes showed increased production of IGF-1, upregulated CD206, and enhanced phagocytosis. These different states of microglial activation may also have neuronal consequences. Endothelial-activated microglia were moderately neurotoxic, whereas astrocyte-activated microglia promoted neuronal dendritic plasticity. Taken together, our present findings may allow one to hypothesize a gliovascular mechanism for regulating the microglial switch (Fig. 6).

Although the present study is based on cell biology model systems, these findings may allow one to propose a new hypothesis with in vivo implications, especially for stroke. Under normal conditions, microglia reside behind the blood-brain barrier. During the acute phase of stroke, the loss of blood-brain barrier (BBB) integrity [37] allows microglia to now “see” the endothelium, resulting in the first state of activation that may be potentially damaging. Thereafter, during stroke recovery, the BBB is re-sealed and astrogliosis begins to take place [38], and now, exposure to reactive astrocytes may switch the microglia into the second state of activation that could be potentially beneficial. Preliminary results may support this hypothesis. Microglia isolated from the ischemic rodent brains showed distinct waves of gene expression (Additional file 3: Figure S3). At 3 days after transient focal ischemia, microglia expressed high levels of TNFα and IL-1β, similar to our endothelial-activated microglial cultures. By 14 days post-ischemia, TNFα and IL-1β levels tended to come back down, whereas the expression of IGF-1 were increased, similar to our astrocyte-activated microglial cultures. Of interest, this biphasic pattern of microglial activation after focal cerebral ischemia seemed to approximately match the well-known timing of BBB breakdown followed by proliferation of reactive astrocytes (Additional file 4: Figure S4).

Microglial activation in the CNS will be complex, and its phenotype may not be cleanly divided into either classically activated “M1-like” states or alternatively activated “M2-like” states. Instead, an entire spectrum of phenotypes may exist that span the range from deleterious to regulatory to remodeling effects [39–42]. Whether certain microglial phenotypes are beneficial or detrimental depends on the situation. Any insult could activate microglia, and the primary purpose of microglial activation is to help. Microglial migration and phagocytosis may be good for the removal of infective pathogens and dead neurons after injury. However, overactivated microglia may further damage neurons. Our study supported this idea. Endothelium-activated microglia had typical amoeboid morphology, but they had a reduced ability to phagocytose. Microglia activated by endothelium not only released a large amount of pro-inflammatory factors, such as TNFα and IL-1β, but also increased the release of anti-inflammatory IL-10 at the same time. Astrocyte-activated microglia showed enhanced phagocytosis with non-amoeboid shape and upregulated the “M2-like” marker CD206, but also slightly increased the “M1-like” marker iNOS. Hence, a simple “M1-like” versus “M2-like” classification may not be appropriate for microglial phenotyping after stroke. However, if we consider the classification of microglial phenotype according to their functions, our findings may suggest that endothelial cells somehow activated microglia into a potentially damaging phenotype—toxic to neurons. In contrast, astrocytes somehow induced a different activation state in microglia—promoting neuroplasticity.

Different transcription factors may play key roles in microglial regulation, including members of the IRF and STAT families [23]. A preliminary assessment in our model system suggests that neither endothelium nor astrocytes altered STAT1, STAT3, or STAT6, but significant changes were noted for the IRF family. Endothelial-activated microglia downregulated IRF4 and IRF5 but upregulated IRF7. In astrocyte-activated microglia, IRF4, IRF5, and IRF7 were all uniformly downregulated. IRFs comprise a group of transcription factors that are related to the regulation of gene expression in the immune response. IRF4 is a negative regulator of inflammation and mediates M2 polarization of macrophages [43–45]. IRF5 induces the expression of pro-inflammatory factors and promotes the M1 polarization of macrophages [46, 47]. More recently, it has been proposed that the balance between IRF5 and IRF4 influences microglial activity and corresponds to infarct size and neurological deficits [48, 49]. In our model system, IRF5/IRF4 ratios were significantly higher in endothelial-activated microglia compared to astrocyte-activated microglia, consistent with potentially deleterious properties of the former versus the latter. The role of IRF7 in microglial polarization may be controversial. One study showed that IRF7 expression increased during M2-like to M1-like conversions in microglia, and knockdown of IRF7 suppressed the expression of M1-like markers induced by LPS [50]. Another study found that the decrease of IRF7 kept microglia in M1-like polarization and upregulation of IRF7 switched microglia to M2-like phenotype [51]. In our study, exposure to OGD-injured endothelial media upregulated IRF7 in recipient microglia, suggesting that IRF7 may be related to an initially damaging phenotype. Gain- and loss-of-function experiments will be required to carefully define the role of IRFs in the gliovascular switch for microglia.

Taken together, our findings suggest that differential signaling from injured endothelium versus astrocytes may underlie a potential gliovascular mechanism for microglial regulation and polarization. However, regardless of any cell-autonomous crosstalk, microglia can possibly respond to OGD by itself and be activated without other cell stimulation. To clarify this issue, we included two more experiments. At first, we detected gene expression and cytokine release in OGD-treated microglia (Additional file 5: Figure S5). mRNA levels of iNOS, CD86, CD206, and arginase 1 did not show significant changes in microglia subjected to OGD for 4 h and reoxygenation for 4 h. In addition, compared with normoxic microglia, OGD did not significantly change the microglial release of IGF-1, IL-10, IL-1β, and TNFα. So OGD alone may not significantly affect the characteristics and phenotypes of microglia. Next, we detected gene expression and cytokine release of endo-CM- or astro-CM-treated microglia that were previously subjected to OGD for 4 h before media transfer (Additional file 6: Figure S6). The expression of iNOS, CD206, and arginase 1 in OGD-treated microglia showed similar changes as normoxic microglia, although there were slight differences in CD86 in OGD-treated microglia. However, the changes of TNFα and IL-10 release from OGD-treated microglia were similar as normoxic microglia. Overall, although there were slight differences, it seemed that the responses of OGD-treated microglia to endo-CM or astro-CM might be similar to normoxic microglia in most aspects, suggesting that endothelial and astrocytic crosstalk may still be relevant in the context of overall tissue OGD.

There are several caveats for the present study. First, our data suggested that endothelium and astrocyte might play different roles in microglia phenotypic polarization after ischemic stroke. However, we do not know the factors in endothelial versus astrocyte secretomes that are responsible for differential signaling. It is likely that combinations of factors will be involved. We will further explore the components inside the conditioned media that may be responsible for differential microglial activation in future studies. Second, our experiments are based on media transfer, so signaling is restricted to released and diffusible mediators. However, within an intact neurovascular unit, direct cell-cell and cell-matrix interactions should also play a role. Third, we only measured selected cytokines and genes that were known to play a role in microglial regulation. A full construction of the microglial transcriptome under different activation states will be eventually required. Fourth, we used the RBE4 cell line in our present study instead of primary cultured endothelial cells. RBE4 cells are a commonly used immortalized rat brain microvessel endothelial cell line that is very commonly used to study neurovascular physiology as well as BBB function in vitro [52]. RBE4 cells exhibit a non-transformed phenotype, possess reproducible sets of endothelial cell markers, and express BBB-specific properties [52]. Compared with RBE4 cells, primary culture of brain endothelial cells is time-consuming and expensive and is difficult to eliminate all non-endothelial cell contaminants. In addition, primary cultured endothelial cells rapidly de-differentiate in vitro and lose the characteristics of BBB endothelial cells after a few passages in culture [52]. The present study used RBE4 cells in order to establish proof of concept for our new hypothesis. Further studies should consider using primary endothelial cells for repeating key experiments. Fifth, immune response after ischemia in the brain is complicated. Besides the activation of resident immune cell microglia, the infiltration of leukocytes into the brain parenchyma following ischemic stroke is well-established, and the infiltrated immune cells comprise both innate (e.g., neutrophils, macrophages) and subsequently adaptive (e.g., T and B cells) immune systems [53]. The interaction between the infiltrated peripheral immune cells and macrophages may affect the status of microglial phenotypes and should be considered carefully in future studies.

## Conclusions

This proof of concept study may allow one to hypothesize a differential endothelial versus astrocyte mechanism for activating microglia. In vivo studies are warranted to rigorously assess and map this gliovascular switch for microglia in stroke and other CNS disorders.

## Additional files

Additional file 1: **Figure S1.** Cell viability of endothelial cells and astrocytes after OGD for 4 h and reoxygenation for 24 h. (PDF 56 kb)

Additional file 2: **Figure S2.** Levels of TNFα, IL-1β, IL-10, and IGF-1 in culture media from OGD-treated endothelial cells (Endo) or astrocytes (Astro) were measured using ELISA. (PDF 94 kb)

Additional file 3: **Figure S3.** Gene expression of microglia sorted from the ischemic brains by FACS was detected using real-time PCR at different time points after ischemia. (PDF 75 kb)

Additional file 4: **Figure S4.** (a) IgG immunostaining of the brain sections of rat transient MCAo models at 1, 3, and 7 days after ischemia. Data were expressed as mean ± SEM. *p < 0.05 (one-way ANOVA). (b) GFAP immunostaining of the brain sections of rat transient MCAo models at 1, 3, and 7 days after ischemia. (PDF 3094 kb)

Additional file 5: **Figure S5.** Gene expression and cytokine release from OGD-treated microglia. Microglia were subjected to OGD for 4 h and reoxygenation for 4 h (for gene expression detection using real-time PCR) or 24 h (for cytokine release detection using ELISA). (a) The levels of iNOS, CD86, CD206, and arginase1 did not significantly change in microglia after OGD treatment. (b) Compared with normoxic microglia, OGD treatment did not significantly change the release of IGF-1, IL-10, IL-1β, and TNFα from microglia. (PDF 74 kb)

Additional file 6: **Figure S6.** Microglia were subjected to OGD for 4 h, and then endo-CM or astro-CM were added into OGD-treated microglia for another 8 h (for gene expression experiment using real-time PCR) or 24 h (for ELISA measurement of cytokines). (a) Endo-CM treatment upregulated the expression of iNOS, whereas astro-CM treatment upregulated the expression of CD206. (b, c) Endo-CM significantly increased the release of TNFα and IL-10 from OGD-treated microglia. (PDF 104 kb)

## Abbreviations

ANOVA: Analysis of variance
BBB: Blood-brain barrier
CD: Cluster of differentiation
CNS: Central nervous system
DMEM: Dulbecco’s modified Eagle’s medium
ELISA: Enzyme-linked immunosorbent assay
FBS: Fetal bovine serum
IGF-1: Insulin-like growth factor 1
IL: Interleukin
iNOS: Inducible nitric oxide synthase
IRF: Interferon regulatory factor
MHC: Major histocompatibility complex
MTT: 3-(4,5-Dimethylthiazol-2-Yl)-2,5-diphenyltetrazolium bromide
OGD: Oxygen-glucose deprivation
SD: Standard deviation
SEM: Standard error of mean
STAT: Signal transducer and activator of transcription
TNF: Tumor necrosis factor
