# Supplementary material for: A potential gliovascular mechanism for microglial activation: differential phenotypic switching of microglia by endothelium versus astrocytes
Source: J Neuroinflammation. 2018 May 15;15:143. doi: 10.1186/s12974-018-1189-2 (PMC5952884; doi:10.1186/s12974-018-1189-2)

Additional file 1: Figure S1: Cell viability of endothelial cells and astrocytes after OGD for 4 hrs and reoxygenation for 24 hrs.

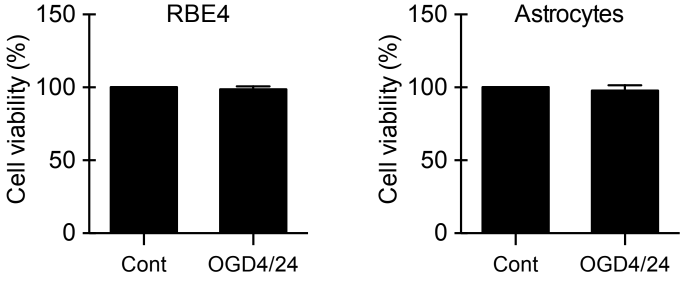

Supplement: Supplementary file 1 — Figure S1. Cell viability of endothelial cells and astrocytes after OGD for 4 h and reoxygenation for 24 h. (PDF 56 kb) [file 12974_2018_1189_MOESM1_ESM.pdf]
