# Supplementary material for: A potential gliovascular mechanism for microglial activation: differential phenotypic switching of microglia by endothelium versus astrocytes
Source: J Neuroinflammation. 2018 May 15;15:143. doi: 10.1186/s12974-018-1189-2 (PMC5952884; doi:10.1186/s12974-018-1189-2)

Additional file 2: Figure S2: Levels of TNFα, IL-1β, IL-10 and IGF-1 in culture media from OGD-treated endothelial cells (Endo) or astrocytes (Astro) were measured using ELISA.

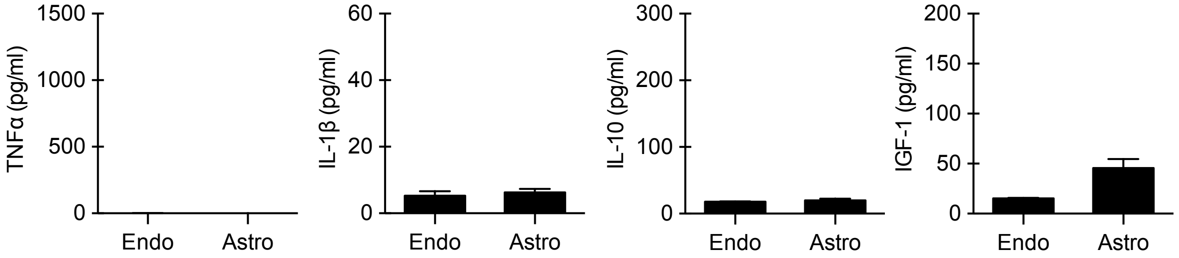

Supplement: Supplementary file 2 — Figure S2. Levels of TNFα, IL-1β, IL-10, and IGF-1 in culture media from OGD-treated endothelial cells (Endo) or astrocytes (Astro) were measured using ELISA. (PDF 94 kb) [file 12974_2018_1189_MOESM2_ESM.pdf]
