# Supplementary material for: A potential gliovascular mechanism for microglial activation: differential phenotypic switching of microglia by endothelium versus astrocytes
Source: J Neuroinflammation. 2018 May 15;15:143. doi: 10.1186/s12974-018-1189-2 (PMC5952884; doi:10.1186/s12974-018-1189-2)

Additional file 3: Figure S3: Gene expression of microglia sorted from ischemic brains by FACS was detected using real-time PCR at different time points after ischemia.

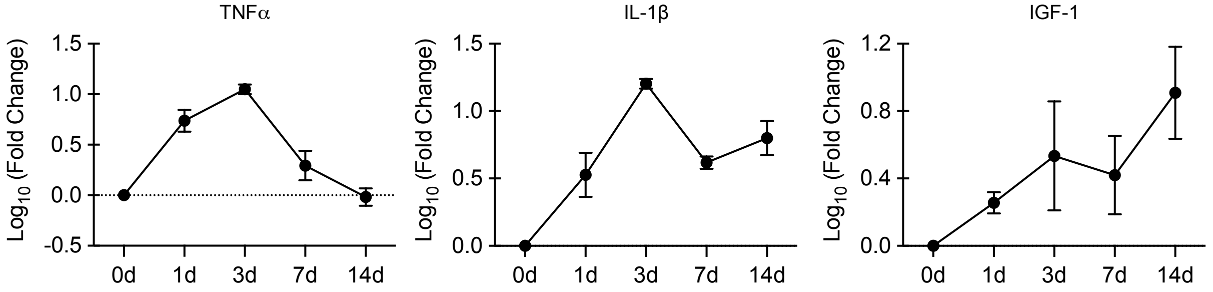

Supplement: Supplementary file 3 — Figure S3. Gene expression of microglia sorted from the ischemic brains by FACS was detected using real-time PCR at different time points after ischemia. (PDF 75 kb) [file 12974_2018_1189_MOESM3_ESM.pdf]
