# Supplementary material for: A potential gliovascular mechanism for microglial activation: differential phenotypic switching of microglia by endothelium versus astrocytes
Source: J Neuroinflammation. 2018 May 15;15:143. doi: 10.1186/s12974-018-1189-2 (PMC5952884; doi:10.1186/s12974-018-1189-2)

Additional file 4: Figure S4: (a) IgG immunostaining of brain sections of rat transient MCAo models at 1, 3, and 7 days after ischemia. Data were expressed as mean±SEM. *, $p<0.05$ (one-way ANOVA). (b) GFAP immunostaining of brain sections of rat transient MCAo models at 1, 3, and 7 days after ischemia.

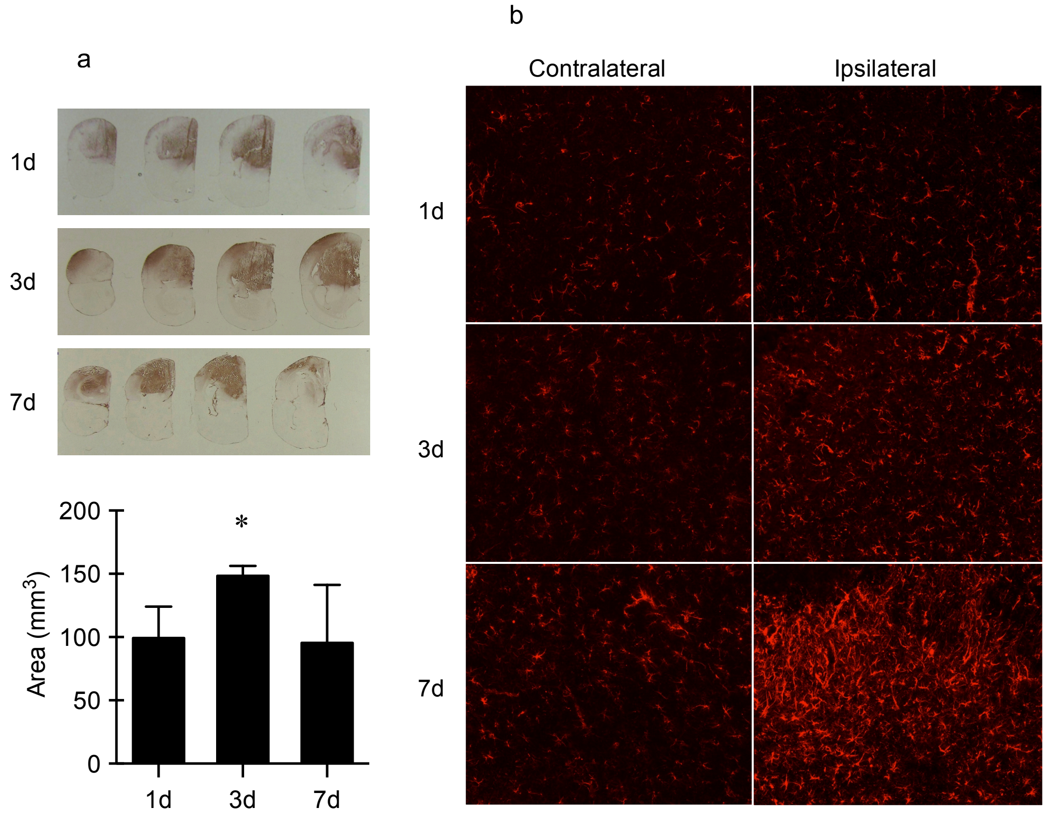

Supplement: Supplementary file 4 — Figure S4. (a) IgG immunostaining of the brain sections of rat transient MCAo models at 1, 3, and 7 days after ischemia. Data were expressed as mean ± SEM. *p < 0.05 (one-way ANOVA). (b) GFAP immunostaining of the brain sections of rat transient MCAo models at 1, 3, and 7 days after ischemia. (PDF 3094 kb) [file 12974_2018_1189_MOESM4_ESM.pdf]
