# Supplementary material for: A potential gliovascular mechanism for microglial activation: differential phenotypic switching of microglia by endothelium versus astrocytes
Source: J Neuroinflammation. 2018 May 15;15:143. doi: 10.1186/s12974-018-1189-2 (PMC5952884; doi:10.1186/s12974-018-1189-2)

Additional file 5: Figure S5: Gene expression and cytokine release from OGD-treated microglia. Microglia were subjected to OGD for 4 hrs and reoxygenation for 4 hrs (for gene expression detection using real time PCR) or 24 hrs (for cytokine release detection using ELISA). (a) The levels of iNOS, CD86, CD206, and arginase1 did not significantly changed in microglia after OGD treatment. (b) Compared with normoxic microglia, OGD treatment did not significantly changed the release of IGF-1, IL-10, IL-1β, and TNFα from microglia.

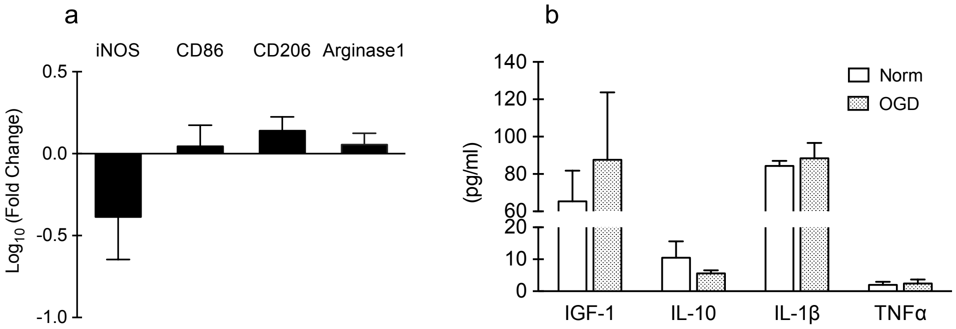

Supplement: Supplementary file 5 — Figure S5. Gene expression and cytokine release from OGD-treated microglia. Microglia were subjected to OGD for 4 h and reoxygenation for 4 h (for gene expression detection using real-time PCR) or 24 h (for cytokine release detection using ELISA). (a) The levels of iNOS, CD86, CD206, and arginase1 did not significantly change in microglia after OGD treatment. (b) Compared with normoxic microglia, OGD treatment did not significantly change the release of IGF-1, IL-10, IL-1β, and TNFα from microglia. (PDF 74 kb) [file 12974_2018_1189_MOESM5_ESM.pdf]
