# Supplementary material for: A potential gliovascular mechanism for microglial activation: differential phenotypic switching of microglia by endothelium versus astrocytes
Source: J Neuroinflammation. 2018 May 15;15:143. doi: 10.1186/s12974-018-1189-2 (PMC5952884; doi:10.1186/s12974-018-1189-2)

Additional file 6: Figure S6: Microglia were subjected to OGD for 4 hrs, and then endo-CM or astro-CM were added into OGD-treated microglia for another 8 hrs (for gene expression experiment using real time PCR) or 24 hrs (for ELISA measurement of cytokines). (a) Endo-CM treatment upregulated the expression of iNOS, whereas astro-CM treatment upregulated the expression of CD206. (b, c) Endo-CM significantly increased the release of TNFα and IL-10 from OGD-treated microglia.

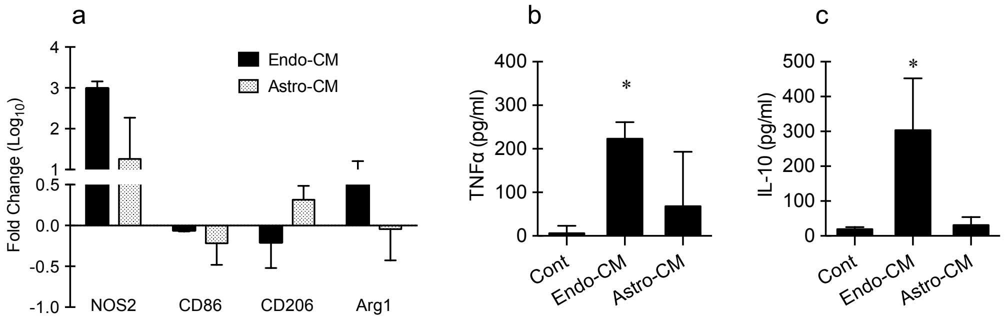

Supplement: Supplementary file 6 — Figure S6. Microglia were subjected to OGD for 4 h, and then endo-CM or astro-CM were added into OGD-treated microglia for another 8 h (for gene expression experiment using real-time PCR) or 24 h (for ELISA measurement of cytokines). (a) Endo-CM treatment upregulated the expression of iNOS, whereas astro-CM treatment upregulated the expression of CD206. (b, c) Endo-CM significantly increased the release of TNFα and IL-10 from OGD-treated microglia. (PDF 104 kb) [file 12974_2018_1189_MOESM6_ESM.pdf]
